# Dolutegravir Plus Two Nucleoside Reverse Transcriptase Inhibitors versus Efavirenz Plus Two Nucleoside Reverse Transcriptase Inhibitors As Initial Antiretroviral Therapy for People with HIV: A Systematic Review

**DOI:** 10.1371/journal.pone.0162775

**Published:** 2016-10-13

**Authors:** George W. Rutherford, Hacsi Horvath

**Affiliations:** Global Health Sciences, University of California, San Francisco, San Francisco, California, United States of America; Universidad Autonoma de Madrid Centro de Biologia Molecular Severo Ochoa, SPAIN

## Abstract

**Background:**

Dolutegravir (DTG) is a once-daily unboosted second-generation integrase-inhibitor that along with two nucleoside reverse transcriptase inhibitors is one of several regimens recommended by the United States, United Kingdom and European Union for first-line antiretroviral treatment of people with HIV infection. Our objective was to review the evidence for the efficacy and safety of DTG-based first-line regimens compared to efavirenz (EFV)-based regimens.

**Methods:**

We conducted a systematic review. We comprehensively searched a range of databases as well as conference abstracts and a trials registry. We used Cochrane methods in screening and data collection and assessed each study’s risk of bias with the Cochrane tool. We meta-analyzed data using a fixed-effects model. We used GRADE to assess evidence quality.

**Results:**

From 492 search results, we identified two randomized controlled trials, reported in five peer-reviewed articles and one conference abstract. One trial tested two DTG-based regimens (DTG + abacavir (ABC) + lamivudine (3TC) or DTG + tenofovir + emtricitabine) against an EFV-based regimen (EFV+ ABC+3TC). The other trial tested DTG+ABC+3TC against EFV+ABC+3TC. In meta-analysis, DTG-containing regimens were superior to EFV-containing regimens at 48 weeks and at 96 weeks (RR = 1.10, 95% CI 1.04–1.16; and RR = 1.12, 95% CI 1.04–1.21, respectively). In one trial, the DTG-containing regimen was superior at 144 weeks (RR = 1.13, 95% CI 1.02–1.24). DTG-containing regimens were superior in reducing treatment discontinuation compared to those containing EFV at 96 weeks and at 144 weeks (RR = 0.27, 95% CI 0.15–0.50; and RR = 0.28, 95% CI 0.16–0.48, respectively). Risk of serious adverse events was similar in each regimen at 96 weeks (RR = 1.15, 95% CI 0.80–1.63) and 144 weeks (RR = 0.93, 95% CI 0.68–1.29). Risk of bias was moderate overall, as was GRADE evidence quality.

**Conclusions:**

DTG-based regimens should be considered in future World Health Organization guidelines for initial HIV treatment.

## Introduction

Dolutegravir (DTG) is a once-daily unboosted second-generation integrase-inhibitor [1,2] that along with two nucleoside reverse transcriptase inhibitors (NRTI) is the third agent in two of the United States (U.S.) Department of Health and Human Services’ and the European AIDS Clinical Society’s six recommended initial regimens for antiretroviral-naïve HIV-infected patients [3,4]. The British HIV Medical Association has also recommended it as one of six third agents to be used with a two-drug NRTI backbone [5]. DTG has a very low resistance profile and a low risk of drug-drug interactions and is available in a fixed dose combination (with abacavir [ABC] and lamivudine [3TC]). Unlike elvitegravir (EVG) [6,7], another integrase inhibitor, DTG does not require boosting. DTG’s efficacy has been evaluated in five Phase IIb, III and IIIb trials, which have involved 1,579 patients [8]. In four studies, participants were ART-naïve (SPRING-1 [9], SPRING-2 [10], SINGLE [11] and FLAMINGO [12]); in one study, participants were ART-experienced but integrase inhibitor-naïve (SAILING) [13].

In contrast to U.S., European and British recommendations, current World Health Organization (WHO) guidelines call for initial therapy with two NRTIs, tenofovir disoproxil fumarate (TDF) and either 3TC or emtricitabine (FTC), plus the non-nucleoside reverse transcriptase inhibitor (NNRTI) efavirenz (EFV) as the preferred regimen in non-pregnant and non-breastfeeding adults [14]. However, EFV in particular has a less than ideal toxicity profile, which primarily includes neuropsychiatric symptoms in up to 50% of patients ranging from dizziness, insomnia and abnormal dreams to depression and suicide [15,16]. For this reason, it has been replaced with integrase inhibitors and the boosted protease inhibitor darunavir/ritonavir for first-line therapy in many high-income countries [3–5]; these regimens have the added benefit of reducing viral load more rapidly. Based on head-to-head comparisons with the original licensed integrase inhibitor, raltegravir, DTG appears to be non-inferior in one trial with ART-naïve patients [10] and superior in terms of efficacy and non-discontinuation in another [12]. WHO lists initial therapy with TDF + 3TC + DTG or TDF + FTC + DTG as an alternative first-line regimen, while noting that safety and efficacy data on its use in pregnant women, people coinfected with HIV and TB and children <12 years old are unavailable [14].

We anticipate that in the next round of WHO recommendations, serious consideration will be given to broadening recommendations for first-line ART to include DTG and possibly darunavir/ritonavir. Part of the WHO process requires carefully conducted systematic reviews to determine the extent and strength of the evidence that can support such a recommendation. In this paper we systematically review the efficacy and safety of DTG in combination with two NRTIs compared to the current WHO standard regimen of EFV with two NRTIs. The two NRTI backbones that we examine include ABC/3TC/DTG and TDF/3TC or FTC plus DTG.

## Methods

We used Cochrane Collaboration methods [17] throughout the review process and follow the Preferred Reporting Items for Systematic Reviews and Meta-Analyses (PRISMA) guidance [18] in reporting our review. We registered our review protocol in the PROSPERO online registry (registration number CRD42014013233).

### Search methods

We formulated a comprehensive and exhaustive search strategy in an effort to identify all relevant studies. We searched the Cochrane Central Register of Controlled Trials, Embase, Literatura Latino Americana em Ciências da Saúde (LILACS), PubMed and Web of Science. Our search strategy included Medical Subject Heading (MeSH) terms and a range of relevant keywords and covered all records up to the search date (16 March 2016). See **S1 Table** for our PubMed search strategy, which we modified and adapted as needed for use in the other databases.

We also searched all available abstracts from the Conference on Retroviruses and Opportunistic Infections, the International AIDS Conference, and the International AIDS Society Conference on HIV Pathogenesis, Treatment and Prevention through March 2016. We searched for ongoing trials in the clinical trials registry (clinicaltrials.gov) at the U.S. National Institutes of Health. Our searches were iterative, in that we searched bibliographies of included and other highly relevant studies for additional references. Studies published in any language were eligible for inclusion. Publication status was not an eligibility criterion.

### Inclusion and exclusion criteria

We included RCTs that compared clinical and laboratory outcomes in HIV-1-infected, ART-naïve adult patients starting regimens of DTG plus two NRTIs with those who started on regimens of EFV plus two NRTIs. We excluded non-randomized studies, studies in which participants were ART-experienced and studies in which DTG was compared to boosted PI regimens.

### Data extraction

We imported search results into bibliographic citation management software (EndNote X7, Thomson Reuters, New York, New York, USA) and excluded duplicate references. Reviewing only article titles, one author (HH) excluded all references that were clearly irrelevant. Two authors (GWR and HH), working independently, then reviewed the titles, abstracts and descriptor terms of the remaining citations to identify potentially eligible reports. We obtained full text articles for all references identified as potentially meeting inclusion criteria. GWR and HH reviewed these full text articles and applied inclusion criteria to establish each study's eligibility or ineligibility. Our plan was to resolve any differences of opinion through discussion and, if necessary, a neutral third party arbiter.

After identifying trials for inclusion, two authors (GWR and HH) independently examined and extracted data from each study. GWR and HH separately entered these data into standardized data extraction forms and then compared extracted data. There were no disagreements.

### Risk of bias assessment

We used the Cochrane Collaboration tool for assessing risk of bias in the included RCTs [17]. The Cochrane tool assesses risk of bias in individual studies in six domains: sequence generation, allocation concealment, blinding, incomplete outcome data, selective outcome reporting and other potential biases.

### Data synthesis and analysis

We assessed efficacy using the relative risk (RR) for dichotomous outcomes and mean difference (MD) for continuous outcomes, each with its 95% confidence interval (CI). Where appropriate and possible, we pooled data across studies and estimated summary effect sizes, using a Mantel-Haenszel fixed-effects meta-analytic model. We performed all meta-analyses in Review Manager 5.3 (Cochrane Collaboration, London, UK). Our outcome measures, which were pre-specified in the protocol, included clinical progression, death, viral suppression to non-detectable levels, discontinuation of therapy, immunologic recovery, acquired resistance and Grade III and IV severe adverse reactions. We did not pre-specify specific adverse events.

We present estimates of heterogeneity, determined by the I^2^ statistic. Estimates of I^2^ are interpreted as the percentage of variability in effect estimates due to heterogeneity rather than to chance. We would have conducted sensitivity analyses had it been necessary to investigate heterogeneity in pooled data. We also pre-specified a sub-group analysis to compare NRTI backbones (TDF+FTC or 3TC vs. ABC+3TC) with DTG-based therapy.

We used the GRADE approach to assess the quality of evidence for each outcome across the literature [19]. In GRADE, “quality of evidence” is defined as “the extent of our confidence that the estimates of effect are correct" [17]. The quality rating across studies has four levels: high, moderate, low, or very low. Data from RCTs are initially considered to be of high quality but can be downgraded for any of five reasons: 1) risk of bias; 2) indirectness of evidence; 3) unexplained heterogeneity or inconsistency of results; 4) imprecision of results; or 5) high probability of publication bias. Data from non-RCTs are considered to be of low quality, but can be upgraded for any of three reasons: 1) large magnitude of effect; 2) plausible confounding would increase confidence in an estimated effect; or 3) the presence of a dose-response gradient.

## Results

**Fig 1:**
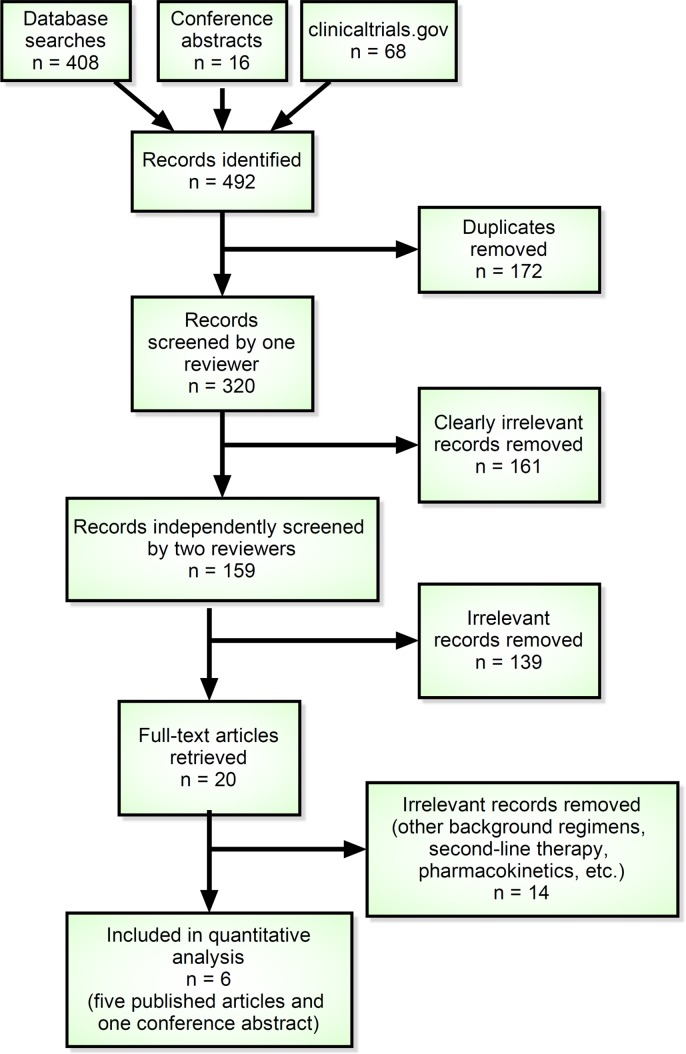
PRISMA flowchart: Flowchart depicting our screening process.

We initially identified 492 articles (bibliographic databases, n = 408; conference abstracts, n = 16; registered trials, n = 68). After removing 172 duplicate records and 161 clearly irrelevant records, we independently reviewed 159 titles and abstracts and excluded 139 clearly irrelevant records. We selected 20 records for full-text review. We then excluded 14 studies reporting results of other background regimens, second-line therapy, pharmacokinetics and other topics. Fig 1 depicts our screening process.

Two trials (reported in five published articles and one conference abstract) met our inclusion criteria. (Table 1). The trials were conducted in Australia, Belgium, Canada, France, Germany, Hungary, Italy, The Netherlands, Romania, Spain, Russia, the United Kingdom and the United States. Overall, there were 934 participants. The first trial (SPRING-1) was a four-arm Phase IIb trial that compared DTG + ABC + 3TC (N = 17) or DTG + TDF + FTC (N = 34) with EFV + either ABC + 3TC (N = 16) or TDF + FTC (N = 34) [9, 20]. The other was a Phase III multicenter trial (SINGLE) that compared DTG + ABC + 3TC with EFV + ABC + 3TC in 833 ART- naïve patients [11,21–23].

**Table 1:** Characteristics of included studies.

Study	Clinicaltrials.gov identifier	Setting	Participants	Intervention	Comparator
SINGLE	NCT01263015	Canada, Denmark, France, Germany, Italy, Netherlands, Romania, Spain, United Kingdom, United States	833	DTG+ABC+3TC	EFV+ABC+3TC
SPRING-1	NCT00951015	France, Germany, Italy, Russia, Spain, United States	101	DTG+ABC+3TC or DTG+TDF+FTC	EFV+ABC+3TC

Legend: DTG, dolutegravir. ABC, abacavir. 3TC, lamivudine. FTC, emtricitabine. TDF, tenofovir.

SPRING-1 contributed 51 patients to the DTG arm and 50 to the EFV arm; SINGLE contributed 414 to the DTG arm and 419 to the EFV arm. Participants in the SPRING-1 trial who received lower doses of DTG (53 randomized to receive 10 mg DTG and 51 randomized to receive 25 mg of DTG daily) were not included in the analysis [20].

**Fig 2:**
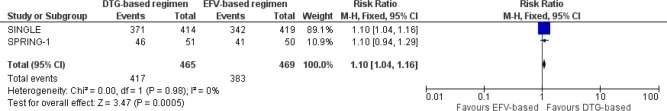
DTG + two NRTI vs. EFV + two NRTIs. Viral suppression to non-detectable (<50 copies/mL) at 48 weeks.

**Fig 3:**
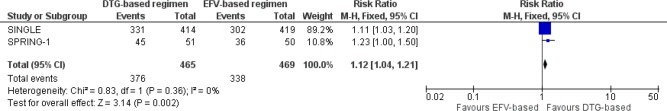
DTG + two NRTI vs. EFV + two NRTIs. Viral suppression to non-detectable (<50 copies/mL) at 96 weeks.

**Fig 4:**
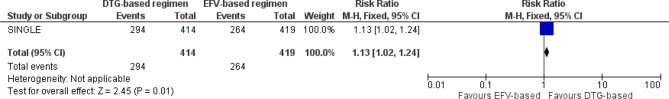
DTG + two NRTI vs. EFV + two NRTIs. Viral suppression to non-detectable (<50 copies/mL) at 144 weeks.

The primary endpoint of these two studies was viral suppression to <50 copies/mL at 48, 96 and 144 weeks. In meta-analysis at each time point, DTG-containing regimens were superior to EFV-containing regimens (RR = 1.10, 95% CI 1.04–1.16 at 48 weeks; RR = 1.12, 95% CI 1.04–1.21 at 96 weeks and RR = 1.13, 95% CI 1.02–1.24 at 144 weeks) (Figs 2–4). Only SINGLE contributed data to the 144-week outcome. There were only two deaths overall (both in the EFV arm in SINGLE) and no difference in risk of death between the two treatment regimens (RR = 0.26, 95% CI 0.01–4.20). SINGLE also reported a primary outcome of discontinuation of initial ART regimen because of adverse events or death at 96 and 144 weeks. At both time points, the DTG-containing regimens were superior to those containing EFV (RR = 0.27, 955 CI 0.15–0.50 at 96 weeks and RR = 0.28, 95% CI 0.16–0.48 at 144 weeks). Risk of serious adverse events was similar in each regimen at 96 weeks (RR = 1.15, 95% CI 0.80–1.63) and 144 weeks (RR = 0.93, 95% CI 0.68–1.29).

The principal secondary outcomes were CD4 recovery and antiretroviral resistance. SINGLE and SPRING-1 contributed data to the 48 and 96-week outcomes; SINGLE alone contributed data to the 144-week outcome. At 48, 96 and 144 weeks immune recovery was significantly more robust among patients taking the DTG-based regimen (+57.9 cells/μL, 95% CI +40.1 to +75.8; +42.2 cells/μL, 95% CI +16.6 to +67.9; and +46.9 cells/μL, 95% CI +15.6 to +78.2, respectively). There was no integrase inhibitor resistance at 96 weeks in either study but 10 instances of NRTI or NNRTI resistance at 96 weeks (RR = 0.09, 95% CI 0.01–0.71). Tables 2 and 3 show results for all outcomes.

Our assessments for heterogeneity with the I^2^ statistic found no (0%) heterogeneity in any meta-analysis of primary or secondary outcomes.

**Table 2:** Dichotomous outcomes, DTG vs. EFV plus two NRTIs for first-line ART.

Outcomes	Studies	RR (95% CI)	Events (DTG)	Total (DTG)	Events (EFV)	Total (EFV)
**Viral suppression to non-detectable (<50 copies/mL) at 48 weeks (pooled data, SINGLE and SPRING-1)**	2	1.10 (1.04 to 1.16)	417	465	383	469
**Viral suppression to non-detectable (<50 copies/mL) at 96 weeks (pooled data, SINGLE and SPRING-1)**	2	1.12 (1.04 to 1.21)	376	465	338	469
**Viral suppression to non-detectable (<50 copies/mL) at 144 weeks (SINGLE)**	1	1.13 (1.02 to 1.24)	294	414	264	419
**Mortality at 48 weeks (pooled data, SINGLE and SPRING-1)**	2	0.2 (0.01 to 4.20)	0	465	2	469
**Discontinuation due to adverse events or death at 96 weeks (SINGLE)**	1	0.27 (0.15 to 0.50)	13	414	48	419
**Discontinuation due to adverse events or death at 144 weeks (SINGLE)**	1	0.28 (0.16 to 0.48)	16	414	58	419
**Clinical disease progression at 48 weeks (pooled data, SINGLE and SPRING-1)**	2	0.83 (0.45 to 1.52)	18	465	22	469
**INI resistance mutation at 96 weeks (pooled data, SINGLE and SPRING-1)**	2	No events	0	465	0	469
**NRTI or NNRTI resistance mutation at 96 weeks (pooled data, SINGLE and SPRING-1)**	2	0.09 (0.01 to 0.71)	0	465	10	469
**Serious adverse events at 96 weeks (pooled data, SINGLE and SPRING-1)**	2	1.15 (0.8 to 1.63)	58	465	51	469
**Serious adverse events at 144 weeks (SINGLE)**	1	0.93 (0.68 to 1.29)	60	414	65	419

Legend: RR, risk ratio; CI, confidence interval; DTG, dolutegravir-based regimen; EFV, efavirenz-based regimen.

**Table 3:** Continuous outcomes, DTG vs. EFV plus two NRTIs for first-line ART.

Outcomes	Studies	MD (95% CI)	Participants (DTG)	Participants (EFV)
**Immunologic recovery (CD4 count Δ) at 48 weeks (pooled data, SINGLE and SPRING-1)**	2	+57.93 (+40.11 to +75.75)	465	469
**Immunologic recovery (CD4 count Δ) at 96 weeks (pooled data, SINGLE and SPRING-1)**	2	+42.21 (+16.62 to +67.81)	465	469
**Immunologic recovery (CD4 count Δ) at 144 weeks (SINGLE)**	1	+46.9 (+15.56 to +78.24)	414	419

Legend: MD, mean difference; CI, confidence interval; DTG, dolutegravir-based regimen; EFV, efavirenz-based regimen.

### Subgroup analysis

SPRING-1 also compared two separate NRTI backbones with DTG and found virologic non-response at 48 weeks in one patient in the DTG+TDF+FTC arm and one in the DTG+ABC+3TC arm (not counting one ABC-arm patient with Burkitt’s lymphoma). Comparing data from SPRING-1’s DTG+TDF+FTC patients (n = 34) with pooled DTG+ABC+3TC data from patients in both the SPRING-1 and SINGLE trials (n = 431), patients receiving DTG+TDF+FTC in SPRING-1 had modestly better virologic response than those receiving DTG+ABC+3TC in either trial (RR = 1.08, 95% CI 1.01–1.16).

### Risk of bias in the included studies

Overall, the risk of bias across both trials was moderate. In both trials, methods for sequence generation were adequate, with centralized, computer-based procedures used to randomize patients within baseline CD4 and viral load strata in SINGLE and within viral load strata and NRTI selection in SPRING-1. Allocation concealment and blinding of patients and personnel was adequate in SINGLE, although at week 96 patients and personnel were unblinded. Outcome assessors were unblinded at week 48. In SPRING-1, however, allocation to drug was not concealed; only drug dose was concealed. Similarly, participants and personnel were blinded only to drug dose. Outcome assessors were blinded to both drug and dose. With all outcomes biologically measured in both trials, the risk of bias from unconcealed allocation or lack of blinding is unclear, though it is likely low.

There was a high risk of attrition bias in SINGLE, with 18% of DTG arm participants and 26% of EFV arm participants leaving the trial by week 96. While clinical reasons for withdrawal up to week 96 are described well, other types of reasons are not clearly described. Withdrawals after week 96 were described even less clearly. In SPRING-1, attrition was low (6% in DTG arm, 10% in EFV arm), and investigators described it adequately. With regard to selective outcome reporting, both trials conformed well to their respective protocols, though one outcome in SINGLE (“Change from baseline in CD4+ cells at week 48”) was reported only the clinicaltrials.gov web site and not in any published report.

Finally, it should be noted that both trials were sponsored by pharmaceutical companies. The risk of bias this brings to the research is unclear. Although in our review we detected no obvious problems attributable to industry involvement, we describe this involvement here. Conflict of interest forms were available for authors of the key papers. In SINGLE, the initial 2013 manuscript [11] was drafted by a named full-time GlaxoSmithKline (GSK) company employee. Four of 14 named authors on that paper [11] were salaried GSK employees. Another named author was on the GSK Board. Nearly all others had received extensive personal consulting fees and other financial and in-kind considerations from GSK, ViiV Healthcare and other pharmaceutical companies. Nearly all authors on a subsequent SINGLE paper [23] were extensively connected as employees, board members and consultants with GSK, ViiV Healthcare and other businesses. The situation in SPRING-1 was similar, with four of 11 named authors on the trial’s key paper [20] serving as salaried GSK employees.

**Fig 5:**
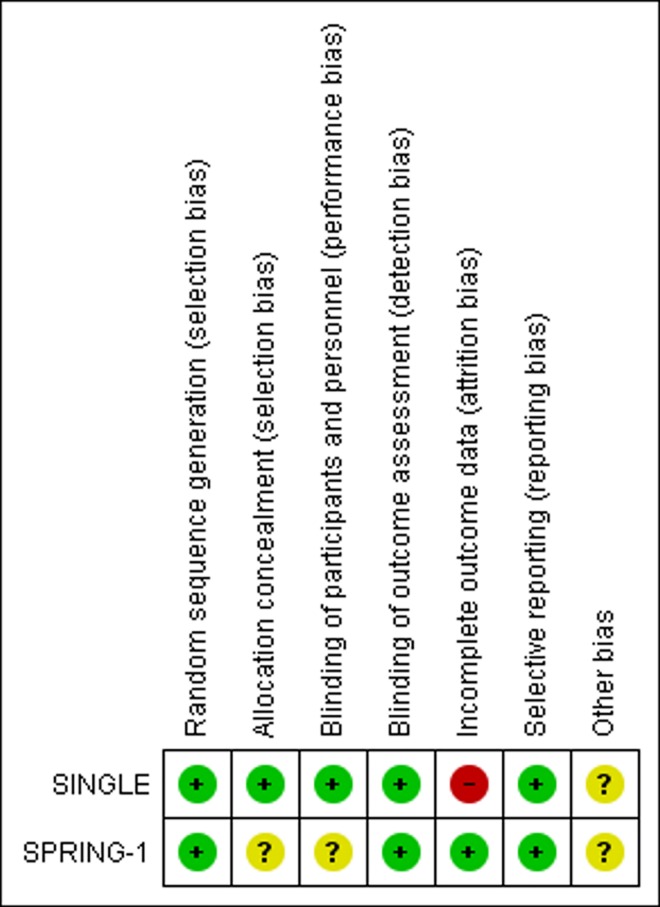
Risk of bias. Review authors' judgments about each risk of bias item for included studies.

Fig 5 provides a summary depiction of bias risk in the included studies. **S2 Table** provides a detailed assessment of bias risk in each trial.

### Quality of the evidence

For the key virologic suppression outcomes, evidence quality was moderate to high. There is high quality evidence from the two trials that virologic suppression was superior at 48 weeks with the DTG-based regimen. At 96 weeks, the two trials provide moderate quality evidence that DTG was superior to EFV. Evidence quality was graded down one level for risk of bias due to the high rate of attrition in SINGLE, a much larger trial than SPRING-1. The 144-week virologic suppression outcome was reported only in SINGLE and was again rated as moderate quality evidence, graded down for risk of bias due to high attrition. There was low quality evidence of no difference in mortality between regimens. Evidence quality was graded down two levels for very serious imprecision (very few events). There was moderate quality evidence from the two trials of no difference between regimens in regard to clinical disease progression at 48 weeks. Evidence quality was graded down one level for serious imprecision (few events).

There was low quality evidence from one trial for strikingly less discontinuation due to adverse events or death at both 96 and 144 weeks. Evidence quality was graded down one level for serious risk of bias (high attrition) and one level for serious imprecision (few events). Evidence quality was not graded up for large effect due to the serious risk of bias and serious imprecision. There was low quality evidence of no difference between regimens in terms of serious adverse events both at 96 weeks (two trials) and at 144 weeks (one trial). Evidence quality was graded down one level for serious risk of bias (high attrition) and one level for serious imprecision (few events).

The two trials contributed high quality evidence of improved immunologic recovery at 48 weeks. One trial provided moderate quality evidence of continued immunologic recovery at 96 and 144 weeks. Evidence quality was graded down one level for risk of bias in these outcomes, due to the high rate of attrition. See **S3 Table** for our complete GRADE evidence profile analysis of evidence quality.

## Discussion

We found that DTG-containing regimens were associated with a greater proportion of patients being virologically suppressed up to 144 weeks after initiation of therapy. We also found that participants in the DTG-containing regimens were almost four-times less likely to discontinue their original regimen because of adverse events or to die than those in the EFV arms. Finally, no participants in the DTG arm developed resistance to integrase inhibitors, while 10 in the EFV arm developed either NRTI or NNRTI resistance.

We also found that patients who were randomized to receive a TDF/FTC backbone were slightly more likely to reach a virologic suppression endpoint than those randomized to receive ABC/3TC. In an exploratory analysis of pooled data from SINGLE and two other trials (SPRING-2 and FLAMINGO, respectively comparing DTG with raltegravir and ritonavir-boosted darunavir in first-line regimens), investigators found no difference between TDF/FTC and ABC/3TC backbones [23]. DTG is co-formulated with ABC+3TC, and for this reason ABC/3TC is the preferred NRTI backbone. Patients initiating ABC+3TC should be screened for predisposition for ABC hypersensitivity reactions with HLA-B*5701 testing if such testing is available [3,4,5]. Also ABC+3TC should be avoided in patients with baseline viral loads >100,000 copies/mL [3,5], but this recommendation has not been made for DTG [3]. On the other hand, TDF should be avoided in patients with osteoporosis and impaired renal function [3,4,5].

WHO’s decisions regarding what therapies to recommend are based on a standardized process that includes multiple inputs [24,25]. The first input is an assessment of efficacy, which includes both a systematic review and an assessment of the quality of the evidence using GRADE evidence profiles. Additional inputs include values and preferences of the intended recipients of the proposed therapy and resource use that adoption of the intervention would require. From these inputs a recommendation is made by a guideline development group to adopt or to not adopt a recommendation and to rate the recommendation as strong or conditional. In this review, we compared DTG-containing first-line regimens with the current standard of EFV plus two NRTIs and assessed the quality of the evidence. Values and preferences and resource use will have to be determined separately in order to proceed with a recommendation.

As with any systematic review our study is limited by the sensitivity of our search and our ability to identify studies that meet our inclusion criteria. We attempted to minimize this risk by comprehensively searching four key databases and hand searching abstracts from three major conferences as well as the bibliographies not only of included articles but also of review articles. Secondly, the two trials on which our conclusions are based were conducted in Australia, Europe and North America; given that the large majority of HIV-infected patients are in Africa and Asia, this may limit the generalizability of our findings. Finally we used the GRADE system to rate the quality of this literature. A recent evaluation of how GRADE is being used at WHO found some remaining challenges [25], but it has emerged as the gold standard for guideline development at WHO [24] and is required by the Guideline Review Committee, which approves all new guidelines.

## Conclusions

We found two RCTs that directly compared DTG and EFV-containing three-drug regimens for initial treatment of HIV infection in adults and adolescents. DTG appears to be superior to EFV in terms of durable viral suppression, absence of resistance and immunologic recovery. DTG-containing regimens should be considered in future international guidelines for initial therapy of HIV infection.

## Supporting Information

S1 Checklist PRISMA reporting checklist. (DOCX)

S1 Table PubMed search strategy, modified and adapted as needed for use in the other databases. (DOCX)

S2 Table Detailed risk of bias assessment. (DOCX)

S3 Table GRADE evidence profile. (DOCX)

## References

[1] Kandel CE, Walmsley SL. Dolutegravir—a review of the pharmacology, efficacy, and safety in the treatment of HIV. Drug Des Devel Ther. 2015;9:3547–55. 10.2147/DDDT.S84850 26185421 PMC4500604

[2] Taha H, Das A, Das S. Clinical effectiveness of dolutegravir in the treatment of HIV/AIDS. Infect Drug Resist. 2015;8:339–52. 10.2147/IDR.S68396 26491363 PMC4598225

[3] Panel on Antiretroviral Guidelines for Adults and Adolescents. Guidelines for the use of antiretroviral agents in HIV-1-infected adults and adolescents. United States Department of Health and Human Services. Available: http://aidsinfo.nih.gov/contentfiles/lvguidelines/AdultandAdolescentGL.pdf.

[4] European AIDS Clinical Society. Guidelines 8.0. October 2015. Available: http://www.eacsociety.org/files/2015_eacsguidelines_8_0-english_rev-20160124.pdf.

[5] British HIV Association (BHIVA). BHIVA guidelines for the treatment of HIV-1-positive adults with antiretroviral therapy 2015. Available: http://www.bhiva.org/documents/Guidelines/Treatment/2015/2015-treatment-guidelines.pdf.

[6] Llibre JM, Pulido F, Garcia F, Garcia Deltoro M, Blanco JL, Delgado R. Genetic barrier to resistance for dolutegravir. AIDS Rev. 2015;17(1):56–64. 25472016

[7] Wainberg MA, Han YS. Will drug resistance against dolutegravir in initial therapy ever occur? Front Pharmacol. 2015;6:90 10.3389/fphar.2015.00090 25972810 PMC4413831

[8] Curtis L, Nichols G, Stainsby C, Lim J, Aylott A, Wynne B, et al Dolutegravir: clinical and laboratory safety in integrase inhibitor-naive patients. HIV Clin Trials. 2014;15(5):199–208. 10.1310/hct1505-199 25350958

[9] Stellbrink HJ, Reynes J, Lazzarin A, Voronin E, Pulido F, Felizarta F, et al Dolutegravir in antiretroviral-naive adults with HIV-1: 96-week results from a randomized dose-ranging study. AIDS. 2013;27(11):1771–8. 10.1097/QAD.0b013e3283612419 23807273 PMC3694319

[10] Raffi F, Rachlis A, Stellbrink HJ, Hardy WD, Torti C, Orkin C, et al Once-daily dolutegravir versus raltegravir in antiretroviral-naive adults with HIV-1 infection: 48 week results from the randomised, double-blind, non-inferiority SPRING-2 study. Lancet. 2013;381(9868):735–43. 10.1016/S0140-6736(12)61853-4 23306000

[11] Walmsley SL, Antela A, Clumeck N, Duiculescu D, Eberhard A, Gutierrez F, et al Dolutegravir plus abacavir-lamivudine for the treatment of HIV-1 infection. N Engl J Med. 2013;369(19):1807–18. 10.1056/NEJMoa1215541 24195548

[12] Clotet B, Feinberg J, van Lunzen J, Khuong-Josses MA, Antinori A, Dumitru I, et al Once-daily dolutegravir versus darunavir plus ritonavir in antiretroviral-naive adults with HIV-1 infection (FLAMINGO): 48 week results from the randomised open-label phase 3b study. Lancet. 2014;383(9936):2222–31. 10.1016/S0140-6736(14)60084-2 24698485

[13] Cahn P, Pozniak AL, Mingrone H, Shuldyakov A, Brites C, Andrade-Villanueva JF, et al Dolutegravir versus raltegravir in antiretroviral-experienced, integrase-inhibitor-naive adults with HIV: week 48 results from the randomised, double-blind, non-inferiority SAILING study. Lancet. 2013;382(9893):700–8. 10.1016/S0140-6736(13)61221-0 23830355

[14] World Health Organization. Consolidated guidelines on the use of antiretroviral drugs for treating and preventing HIV infection: Recommendations for a public health approach—Second edition Geneva: WHO, 6 2016 Available: http://www.who.int/hiv/pub/arv/arv-2016/en/. 27466667

[15] Ford N, Shubber Z, Pozniak A, Vitoria M, Doherty M, Kirby C, et al Comparative Safety and Neuropsychiatric Adverse Events Associated With Efavirenz Use in First-Line Antiretroviral Therapy: A Systematic Review and Meta-Analysis of Randomized Trials. J Acquir Immune Defic Syndr. 2015;69(4):422–9. 10.1097/QAI.0000000000000606 25850607

[16] Shubber Z, Calmy A, Andrieux-Meyer I, Vitoria M, Renaud-Thery F, Shaffer N, et al Adverse events associated with nevirapine and efavirenz-based first-line antiretroviral therapy: a systematic review and meta-analysis. AIDS. 2013;27(9):1403–12. 10.1097/QAD.0b013e32835f1db0 23343913

[17] Higgins JPT, Green S (editors). Cochrane Handbook for Systematic Reviews of Interventions Version 5.1.0 [updated March 2011]. The Cochrane Collaboration, 2011. Available: http://www.cochrane-handbook.org.

[18] Moher D, Liberati A, Tetzlaff J, Altman DG, PRISMA Group. Preferred reporting items for systematic reviews and meta-analyses: the PRISMA statement. PLoS Med. 2009;6(7):e1000097 10.1371/journal.pmed.1000097 19621072 PMC2707599

[19] Guyatt GH, Oxman AD, Schunemann HJ, Tugwell P, Knottnerus A. GRADE guidelines: a new series of articles in the Journal of Clinical Epidemiology. J Clin Epidemiol. 2011;64(4):380–2. 10.1016/j.jclinepi.2010.09.011 21185693

[20] van Lunzen J, Maggiolo F, Arribas JR, Rakhmanova A, Yeni P, Young B, et al Once daily dolutegravir (S/GSK1349572) in combination therapy in antiretroviral-naive adults with HIV: planned interim 48 week results from SPRING-1, a dose-ranging, randomised, phase 2b trial. Lancet Infect Dis. 2012;12(2):111–8. 10.1016/S1473-3099(11)70290-0 22018760

[21] Walmsley S, Berenguer J, Khuong-Josses M-A, et al. Dolutegravir Regimen Statistically Superior to Efavirenz/Tenofovir/Emtricitabine: 96-Week Results From the SINGLE Study (ING114467) [Abstract number 543]. 21st Conference on Retroviruses and Opportunistic Infection, Boston, Massachusetts, 3–6 March 2014.

[22] Raffi F, Rachlis A, Brinson C, Arasteh K, Gorgolas M, Brennan C, et al Dolutegravir efficacy at 48 weeks in key subgroups of treatment-naive HIV-infected individuals in three randomized trials. AIDS. 2015;29(2):167–74. 10.1097/QAD.0000000000000519 25387312 PMC4284010

[23] Walmsley S, Baumgarten A, Berenguer J, Felizarta F, Florence E, Khuong-Josses MA, et al Brief Report: Dolutegravir Plus Abacavir/Lamivudine for the Treatment of HIV-1 Infection in Antiretroviral Therapy-Naive Patients: Week 96 and Week 144 Results From the SINGLE Randomized Clinical Trial. J Acquir Immune Defic Syndr. 2015;70(5):515–9. 10.1097/QAI.0000000000000790 26262777 PMC4645960

[24] World Health Organization. WHO Handbook for Guideline Development 2nd edition Geneva: World Health Organization, 2014 Available: http://www.who.int/kms/guidelines_review_committee/en/.

[25] Sinclair D, Isba R, Kredo T, Zani B, Smith H, Garner P. World Health Organization guideline development: an evaluation. PloS One. 2013;8(5):e63715 10.1371/journal.pone.0063715 23741299 PMC3669321

