# Supplementary material for: Dolutegravir Plus Two Nucleoside Reverse Transcriptase Inhibitors versus Efavirenz Plus Two Nucleoside Reverse Transcriptase Inhibitors As Initial Antiretroviral Therapy for People with HIV: A Systematic Review
Source: PLoS One. 2016 Oct 13;11(10):e0162775. doi: 10.1371/journal.pone.0162775 (PMC5063380; doi:10.1371/journal.pone.0162775)
Supplement: S1 Table — (DOCX) [file pone.0162775.s002.docx]

**S1. PubMed search strategy.** PubMed search strategy, which was modified and adapted as needed for use in the other databases

| **Search** | **PubMed query** |
| --- | --- |
| **#4** | **Search #1 AND #2 AND #3** |
| #3 | Search randomized controlled trial[pt] OR randomized controlled trials[mh] OR random allocation[mh] OR controlled clinical trial[pt] OR randomized[tw] OR randomised[tw] OR randomly[tw] OR random*[tw] OR trial[tiab] OR groups[tiab] |
| #2 | Search HIV Integrase Inhibitors[mh] OR HIV Infections/drug therapy[mh] OR reverse transcriptase inhibitors[mh] OR Antiretroviral Therapy, Highly Active[mh] OR Anti-Retroviral Agents[mh] OR integrase inhibitor*[tw] OR integrase strand transfer inhibitor*[tw] OR INSTI[tw] OR antiretroviral[tw] OR anti-retroviral[tw] OR anti-HIV*[tw] OR anti-AIDS[tw] |
| #1 | Search dolutegravir[tw] OR DTG[tw] OR S/GSK1349572[tw] OR GSK-1349572[tw] OR Tivicay[tw] OR dolutegravir[Supplementary Concept] |

Legend: [mh] = Medical Subject Heading (MeSH) term. [pt] = publication type. [tw] = text word. [tiab] = title or abstract. *= “wildcard”; any character or sequence of characters may follow.
