# Supplementary material for: Dolutegravir Plus Two Nucleoside Reverse Transcriptase Inhibitors versus Efavirenz Plus Two Nucleoside Reverse Transcriptase Inhibitors As Initial Antiretroviral Therapy for People with HIV: A Systematic Review
Source: PLoS One. 2016 Oct 13;11(10):e0162775. doi: 10.1371/journal.pone.0162775 (PMC5063380; doi:10.1371/journal.pone.0162775)
Supplement: S2 Table — (DOCX) [file pone.0162775.s003.docx]

**S2 Table. Risk of bias.** Detailed risk of bias assessment

| **Risk of bias: SINGLE** | **Judgment** | **Rationale** |
| --- | --- | --- |
| Random sequence generation (selection bias) | low | Protocol reports: "The central randomization schedule, including stratification, was generated using the GSK validated randomization software RandAll." Block sizes of six. Centralized procedure. Randomization was stratified according to viral load and CD4 count. |
| Allocation concealment (selection bias) | low | Participants received unique identifying numbers denoting the study arms to which they had been assigned. |
| Blinding of participants and personnel (performance bias) | low | In addition to their assigned regimens, all patients received placebo matching the other regimen's drug, up to week 96. Personnel also blinded up to week 96. Open label after week 96. |
| Blinding of outcome assessment (detection bias) | low | Study was unblinded to outcome assessors for the purpose of the week 48 analysis; participants and personnel remained blinded up to week 96. |
| Incomplete outcome data (attrition bias) | high | Attrition was high by week 96 (DTG arm, 18%; EFV arm, 26%) and even higher by week 144 (DTG arm, 23%; EFV arm, 34%). Clinical reasons for withdrawal are described well at week 96. However, other types of attrition are not well described ("...categories of lost to follow up, withdrew consent, protocol deviations, and investigator discretion were fewer and similar between arms"). After week 96, "One participant in each arm declined to participate in the open-label phase.... Reasons for withdrawal by W144 were similar between treatment arms." Clinical reasons for withdrawal after week 96 are not specifically reported. Supplementary appendix does not add clarity. |
| Selective reporting (reporting bias) | low | Outcomes reported conform well to trial protocol, although "Change from baseline in CD4+ cells at week 48" was only reported on clinicaltrials.gov web site -- not in published literature to date. |
| Other bias | unclear | Industry-funded trial; initial Walmsley 2013 manuscript was drafted by a named full-time GSK employee. Interest disclosure forms are available. Four of 14 named authors on Walmsley 2013 were salaried GSK employees. Another named author was on GSK Board. Nearly all others had received extensive personal consulting fees and other financial and in-kind considerations from GSK, VIIV and other pharmaceutical companies. Nearly all authors on Walmsley 2015 were extensively connected as employees, board members, consultants etc. with GSK, VIIV and other pharmaceutical companies. |
| **Risk of bias: SPRING-1** | **Judgment** | **Rationale** |
| Random sequence generation (selection bias) | low | Randomization was by a central integrated voice-response system according to a computer-generated code and was stratified by VL at screening (≤100 000 copies per mL or >100 000 copies per mL) and baseline NRTI selection. |
| Allocation concealment (selection bias) | high | Only dose was blinded. Participants and personnel were aware of drug allocation. |
| Blinding of participants and personnel (performance bias) | high | Only dose was blinded. "The dose of dolutegravir was masked with matched placebo tablets. Dose but not drug allocation was masked from participants and investigators." Dose was unblinded after week 96. |
| Blinding of outcome assessment (detection bias) | low | "Laboratory analyses were done centrally by Quest Diagnostic Laboratories" |
| Incomplete outcome data (attrition bias) | low | Attrition was light (6%-10%) and investigators described it adequately. |
| Selective reporting (reporting bias) | low | Outcomes reported conform well to trial protocol (http://www.gsk-clinicalstudyregister.com/study/112276#ps) |
| Other bias | unclear | Industry-funded trial. Four of 11 named authors of van Lunzen 2013 were salaried employees of GSK. |
