# Supplementary material for: Dolutegravir Plus Two Nucleoside Reverse Transcriptase Inhibitors versus Efavirenz Plus Two Nucleoside Reverse Transcriptase Inhibitors As Initial Antiretroviral Therapy for People with HIV: A Systematic Review
Source: PLoS One. 2016 Oct 13;11(10):e0162775. doi: 10.1371/journal.pone.0162775 (PMC5063380; doi:10.1371/journal.pone.0162775)
Supplement: S3 Table — (DOCX) [file pone.0162775.s004.docx]

**S3. Quality of the evidence.** GRADE evidence profile

**Author(s):** George W. Rutherford, Hacsi Horvath
**Date:** 2016-04-04
**Question:** Should Dolutegravir 50 mg qd + two NRTIs vs Efavirenz 600 mg qd + two NRTIs be used for initial ART in people with HIV?
**Settings:** Australia, Belgium, Canada, France, Germany, Hungary, Italia, Netherlands, Romania, Spain, Russia, United Kingdom, United States
**Bibliography: SINGLE** trial (Walmsley 2013, Walmsley 2014, Walmsley 2015a, Walmsley 2015b); **SPRING-1** trial (van Lunzen 2012, Stellbrink 2015)

| **Quality assessment** | | | | | | | **No of patients** | | **Effect** | | **Quality** | **Importance** |
| --- | --- | --- | --- | --- | --- | --- | --- | --- | --- | --- | --- | --- |
|  |  |  |  |  |  |  |  |  |  |  |  |  |
| **No of studies** | **Design** | **Risk of bias** | **Inconsistency** | **Indirectness** | **Imprecision** | **Other considerations** | **Dolutegravir 50 mg qd + two NRTIs** | **Efavirenz 600 mg qd + two NRTIs** | **Relative (95% CI)** | **Absolute** |  |  |
| **Viral suppression to non-detectable (<50 copies/mL) at 48 weeks** | | | | | | | | | | | | |
| 2 | randomised trials | no serious risk of bias | no serious inconsistency | no serious indirectness | no serious imprecision | see footnote^4^ | 417/465  (89.7%) | 383/469  (81.7%) | RR 1.1 (1.04 to 1.16) | 82 more per 1000 (from 33 more to 131 more) | ⊕⊕⊕⊕ HIGH | CRITICAL |
| **Viral suppression to non-detectable (<50 copies/mL) at 96 weeks** | | | | | | | | | | | | |
| 2 | randomised trials | serious^1^ | no serious inconsistency | no serious indirectness | no serious imprecision | see footnote^4^ | 376/465  (80.9%) | 338/469  (72.1%) | RR 1.12 (1.04 to 1.21) | 86 more per 1000 (from 29 more to 151 more) | ⊕⊕⊕O MODERATE | CRITICAL |
| **Viral suppression to non-detectable (<50 copies/mL) at 144 weeks** | | | | | | | | | | | | |
| 1 | randomised trials | serious^1^ | no serious inconsistency | no serious indirectness | no serious imprecision | see footnote^4^ | 294/414  (71%) | 264/419  (63%) | RR 1.13 (1.02 to 1.24) | 82 more per 1000 (from 13 more to 151 more) | ⊕⊕⊕O MODERATE | CRITICAL |
| **Mortality at 48 weeks** | | | | | | | | | | | | |
| 2 | randomised trials | no serious risk of bias | no serious inconsistency | no serious indirectness^2^ | very serious^2^ | see footnote^4^ | 0/465  (0%) | 2/469  (0.43%) | RR 0.2 (0.01 to 4.2) | 3 fewer per 1000 (from 4 fewer to 14 more) | ⊕⊕OO LOW | CRITICAL |
| **Discontinuation due to adverse events or death at 96 weeks** | | | | | | | | | | | | |
| 1 | randomised trials | serious^1^ | no serious inconsistency | no serious indirectness | serious^3^ | see footnotes^4,5^ | 13/414  (3.1%) | 48/419  (11.5%) | RR 0.27 (0.15 to 0.5) | 84 fewer per 1000 (from 57 fewer to 97 fewer) | ⊕⊕OO LOW | CRITICAL |
| **Discontinuation due to adverse events or death at 144 weeks** | | | | | | | | | | | | |
| 1 | randomised trials | serious^1^ | no serious inconsistency | no serious indirectness | serious^3^ | see footnotes^4,5^ | 16/414  (3.9%) | 58/419  (13.8%) | RR 0.28 (0.16 to 0.48) | 100 fewer per 1000 (from 72 fewer to 116 fewer) | ⊕⊕OO LOW | CRITICAL |
| **Clinical disease progression (HIV-associated conditions, AIDS or death) at 48 weeks** | | | | | | | | | | | | |
| 2 | randomised trials | no serious risk of bias | no serious inconsistency | no serious indirectness | serious^3^ | see footnote^4^ | 18/465  (3.9%) | 22/469  (4.7%) | RR 0.83 (0.45 to 1.52) | 8 fewer per 1000 (from 26 fewer to 24 more) | ⊕⊕⊕O MODERATE | CRITICAL |
| **INI resistance mutation at 96 weeks** | | | | | | | | | | | | |
| 2 | randomised trials | serious^1^ | no serious inconsistency | no serious indirectness | no serious imprecision | see footnote^4^ | 0/465  (0%) | 0/469  (0%) | not pooled | not pooled | ⊕⊕⊕O MODERATE | CRITICAL |
| **NRTI or NNRTI resistance mutation at 96 weeks** | | | | | | | | | | | | |
| 2 | randomised trials | serious^1^ | no serious inconsistency | no serious indirectness | very serious^2^ | see footnote^4^ | 0/465  (0%) | 10/469  (2.1%) | RR 0.09 (0.01 to 0.71) | 19 fewer per 1000 (from 6 fewer to 21 fewer) | ⊕OOO VERY LOW | CRITICAL |
| **Immunologic recovery (CD4 count Δ) at 48 weeks (Better indicated by higher values)** | | | | | | | | | | | | |
| 2 | randomised trials | no serious risk of bias | no serious inconsistency | no serious indirectness | no serious imprecision | see footnote^4^ | 465 | 469 | - | MD 57.93 higher (40.11 to 75.75 higher) | ⊕⊕⊕⊕ HIGH | CRITICAL |
| **Immunologic recovery (CD4 count Δ) at 96 weeks (Better indicated by higher values)** | | | | | | | | | | | | |
| 2 | randomised trials | serious^1^ | no serious inconsistency | no serious indirectness | no serious imprecision | see footnote^4^ | 465 | 469 | - | MD 42.21 higher (16.62 to 67.81 higher) | ⊕⊕⊕O MODERATE | CRITICAL |
| **Immunologic recovery (CD4 count Δ) at 144 weeks (Better indicated by higher values)** | | | | | | | | | | | | |
| 1 | randomised trials | serious^1^ | no serious inconsistency | no serious indirectness | no serious imprecision | see footnote^4^ | 414 | 419 | - | MD 46.9 higher (15.56 to 78.24 higher) | ⊕⊕⊕O MODERATE | CRITICAL |
| **Serious adverse events at 96 weeks** | | | | | | | | | | | | |
| 2 | randomised trials | serious^1^ | no serious inconsistency | no serious indirectness | serious^3^ | see footnote^4^ | 58/465  (12.5%) | 51/469  (10.9%) | RR 1.15 (0.8 to 1.63) | 16 more per 1000 (from 22 fewer to 69 more) | ⊕⊕OO LOW | CRITICAL |
| **Serious adverse events at 144 weeks** | | | | | | | | | | | | |
| 1 | randomised trials | serious^1^ | no serious inconsistency | no serious indirectness | serious^3^ | see footnote^4^ | 60/414  (14.5%) | 65/419  (15.5%) | RR 0.93 (0.68 to 1.29) | 11 fewer per 1000 (from 50 fewer to 45 more) | ⊕⊕OO LOW | CRITICAL |

^1^ High loss to follow-up in SINGLE by weeks 96 and 144. Graded down by 1.
^2^ Very few events (<50). Optimal information size not met. Graded down by 2.
^3^ Few events (<200). Optimal information size not met. Graded down by 1.
^4^ Extensive industry involvement in both trials. Not graded down for this. We did not detect obvious bias attributable to industry involvement.
^5^ Not graded up for large effect due to serious imprecision.
